# Upshot of Some Bioactive Compounds on Angiogenesis in Retinal Pigment Epithelial Cells

**DOI:** 10.1111/jcmm.70327

**Published:** 2025-01-03

**Authors:** Serkan Sen, Murat Kasikci

**Affiliations:** ^1^ Department of Medical Laboratory Techniques, Ataturk Vocational School of Health Services Afyonkarahisar Health Sciences University Afyonkarahisar Turkey; ^2^ Department of Ophthalmology Muğla Training and Research Hospital Mugla Turkey

**Keywords:** angiogenesis, hesperidin, oleuropein, piperine, proanthocyanidins, retioic acid

## Abstract

Nowadays, the use of monoclonal antibodies to target angiogenic signalling pathways is common, but, unfortunately, the clinical activity of these agents is limited. Thus, the development of approaches targeting multiple pathways for anti‐angiogenic effect will lead to increase the clinical benefit. For this purpose, oleuropein, hesperidin, piperine, proanthocyanidins and retinoic acid, which have previously been proven to be bioactive components, anti‐angiogenic performances were experimentally tested in retinal pigment epithelial cells. Bioactive ingredients were applied to retinal pigment epithelial cells at varying doses for 48 h, and then IC_50_ doses were calculated by MTT analysis. VEGFA and VEGFR1 protein levels were measured by ELISA analysis. Using the RT‐PCR technique, the mRNA expression levels of EGF, EGFR, PDGF, PDGFR‐β and HIF1A were analysed. Among all bioactive compounds, the bevacizumab group showed the best anti‐VEGF activity, followed by the proanthocyanidins and piperine groups. Piperine group showed EGF, PDGF and HIF1A expressions; proanthocyanidins, on the other hand, reduced EGFR, PDGF and HIF1A expressions. Retinoic acid showed an angiogenic effect by increasing VEGFA protein levels and EGF and PDGFR‐β expressions. Although hesperidin increased VEGFA protein levels, it decreased EGF, PDGF, PFGFR‐β and HIF1A expression levels. Among the bioactive components stated in previous studies to have anti‐angiogenic properties, only proanthocyanidins and piperin had anti‐VEGF properties. Considering that angiogenesis does not proceed only through VEGF, this study investigated and reported for the first time the effects of relevant bioactive components on angiogenesis through different mechanisms in retinal pigment epithelial cells.

## 1 Introduction

Pathological angiogenesis occurring in the retina is one of the important findings of diseases that cause vision loss, especially diabetic retinopathy [1]. Treatment methods involving anti‐vascular endothelial growth factor (anti‐VEGF) molecules are utilised for addressing different kinds of significant complications of retinal damage: choroidal neovascularisation and macular edema [2]. Bevacizumab (BVZ) is one of the agents commonly used in clinics for this purpose [3].

Bioactive components are not essential substances for the growth and development of living organisms, like primary metabolites, but they can positively impact health by influencing physiological and cellular activities [4]. The aim of this study was to investigate the potential anti‐angiogenic properties of bioactive components in retinal pigment epithelial cells and compare them with BVZ, which has undisputed anti‐angiogenesis properties as a positive control. For this purpose, Oleuropein (OLP) [5], Hesperidin (HSP) [6], PIP (Piperin) [7], Proanthocyanids (PACs) [8] and Retinoic Acid (RA) [9] which were previously found to have anti‐angiogenesis properties in tumour metastasis studies were included in the study. With the growing popularity of these bioactive compounds, their daily intake as supplements has been steadily increasing. In this regard, the study's aim was to investigate the effects of bioactive compounds taken dietary or as preparations on angiogenesis in RPE cells.

OLP is the main active compound that gives a bitter and pungent taste to the extra virgin olive oil extracted from the plant of *Olea europaea* . It is emphasised that OLP may have a wide range of pharmacological uses with anti‐atherogenic, anti‐inflammatory, antimicrobial, antiviral, lipid‐lowering and blood sugar‐reducing effects [10]. PIP is an alkaloid derived from the black pepper or *Piper nigrum* plant and it is renowned for its antioxidant, anticancer, anti‐inflammatory, antiulcer, antithyroid and antimicrobial properties [11]. PACs are condensed tannins with various pharmacological properties such as anticancer, antioxidant, antidiabetic, antimicrobial and neuroprotective attract attention because they can be obtained cheaply from food processing wastes and agricultural wastes [12]. RA are functional as oligosaccharide carriers in epithelial differentiation and synthesis of glycoproteins [13].

**FIGURE 1:**
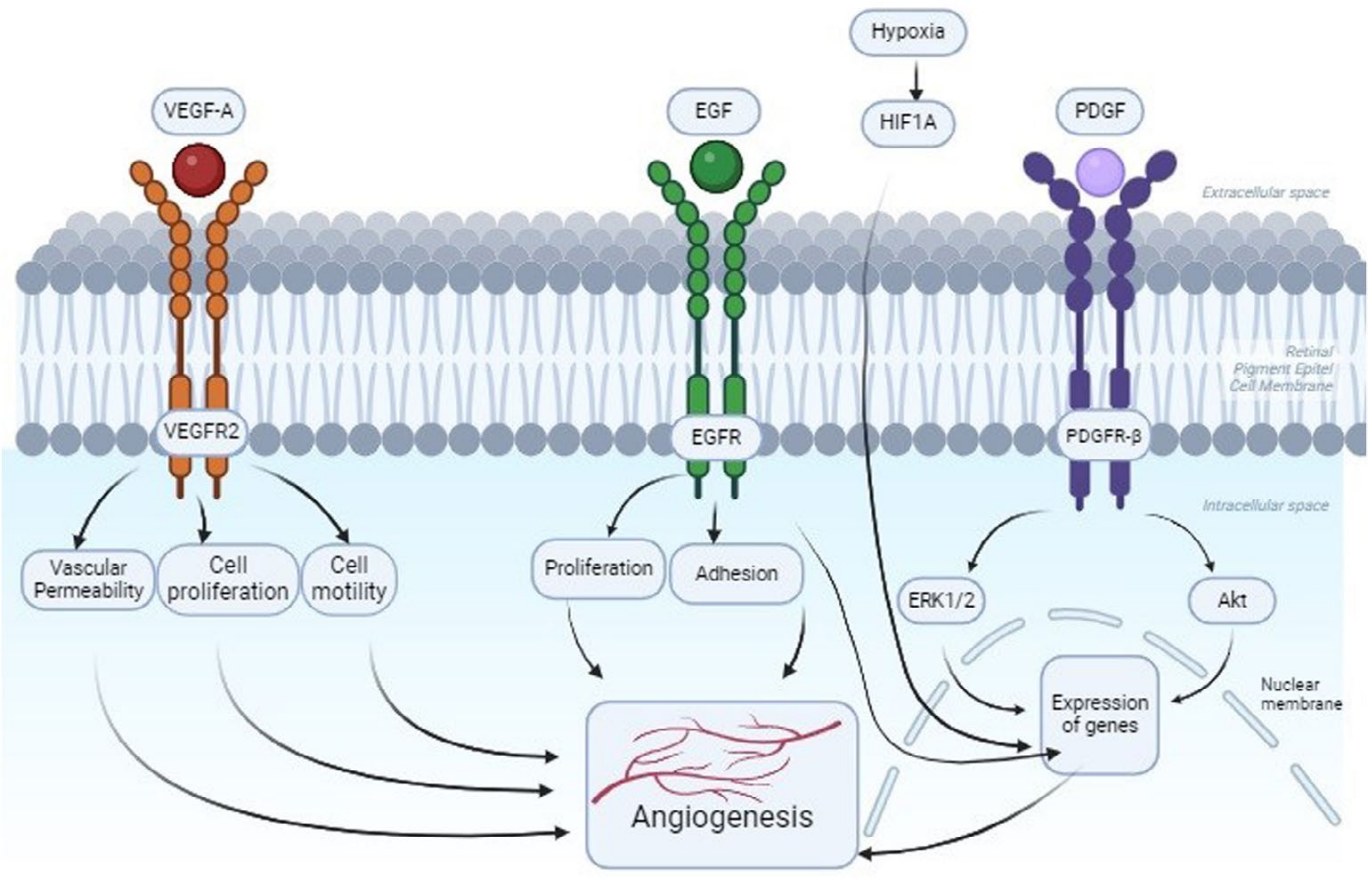
Molecular pathways for angiogenesis in retinal pigment epithelial cells. EGF, Epidermal Growth Factor; EGFR, Epidermal Growth Factor Receptor; HIF1A, Hypoxia‐Inducible Factor‐1α; PDGF, Platelet Platelet‐Derived Growth Factor; PDGFR, Platelet Derived Growth Factor Receptor Beta; VEGFA, Vascular Endothelial Growth Factor A; VEGFR, Vascular Endothelial Growth Factor Receptor.

In recent years, tyrosine kinase inhibitors in small molecules and monoclonal antibodies have been approved to be used as drugs for targeting angiogenic signalling pathways. In general, these agents have shown limited clinical effectiveness, thus increasing the importance of inhibiting multiple pathways. It has been suggested that the two most notable pathways for multi‐pathway targeting will be the EGFR‐VEGFR pathways [14]. Previous studies reported that EGF triggers the signalling cascade of EGF‐EGFR‐MAPK in human RPE cells and plays a crucial role in cohabit the activation of proliferation and migration in RPE cell [15]. It is also known that the development of AMD is triggered when EGFR expression increases [16]. Stimulation of the EGFR pathway enhances secretion of VEGF derived from tumour, which exerts a paracrine effect on endothelial cells, stimulating angiogenesis. Consequently, administration of EGFR inhibitors is typically linked with decreased VEGF expression, whereas resistance to EGFR inhibitors frequently correlates with elevated levels of VEGF. Studies in immunohistochemical conducted on epiretinal membranes isolated from patients with proliferative vitreoretinopathy (PVR) and proliferative diabetic retinopathy (PDR) have shown elevated levels of PDGF and PDGFRα. Moreover, the PDGF derived from platelets was reported to improve migration and proliferation and retinal pigment epithelial (RPE) cells in proliferative vitreoretinopathy (PVR) [17, 18] (Figure 1).

## 2 Material and Method

### 2.1 Reagents and Chemicals

OLP was purchased from Sigma‐Aldrich (Darmstadt, Germany, Cat No: 12247), PIP was procured from Sigma‐Aldrich (Cat No: P49007), HSP was bought from Cayman Chemical (Michigan, USA, Cat No: 18646), PACs was acquired from Biosynth Carbosynth, (Compton, UK, Cat. No: FG34233), RA was obtained from TRC Canada (Toronto, Canada, Cat No: R250000) and BVZ was purchased from Avastin Roche (Basilea, Switzerland). The ARPE‐19 retinal pigment epithelium cell line was obtained from the American Type Culture Collection (ATCC, Manassas, VA, USA; catalogue number CRL‐2302). The VEGFA (Cat No: ELK1129) and VEGFR1 (Cat. No: ELK1126) ELISA kits were purchased from ELK Bioteknology (Denver, USA). All compounds were solubilised in DMSO to a stock concentration of 10 mM and stored at −20°C. Working solutions were freshly prepared by diluting stock solutions in DMEM immediately prior to treatment. All primers were synthesised by Bioligo Biotechnology (Ankara, Türkiye) (Table 1).

**TABLE 1:** Oligonucleotide Primer Sequences and PCR Programs.

Genes	Primer sequences (5′ → 3′)	RT‐PCR programs	Cycle
GAPDH	F‐5′‐GATTTGGTCGTATTGGGCGC 3′ R‐5′‐AGTGATGGCATGGACTGTGG 3′	95°C‐30 s/59°C‐1 m/72°C‐30 s	35
EGF	F‐5′‐CTGAATGTCCCCTGTCCCAC‐3′ R‐5′‐CTCGGTACTGACATCGCTCC‐3′	95°C‐30 s/59°C‐1 m/72°C‐30 s	40
EGFR	F‐5′‐CGCAAAGTGTGTAACGGAATAGG‐3′ R‐5′‐GGCTGACGACTGCAAGAGAA‐3′	95°C‐30 s/58°C‐1 m/72°C‐30 s	40
PDGF‐ß	F‐5′‐CTCGTCCGTCTGTCTCGATG‐3′ R‐5′‐CACACCCACCAAGAGGAGTC‐3′	95°C‐30 s/59°C‐1 m/72°C‐30 s	40
PDGFß‐R	F‐5′‐CACCAACGTGGCTTTTCTGG‐3′ R‐5′‐GGTGCGGTTGTCTTTGAACC‐3′	95°C‐30 s/57°C‐1 m/72°C‐30 s	40
HIF1A	F‐5′‐TGCTGGGGCAATCAATGGAT‐3′ R‐5′‐CTACCACGTACTGCTGGCAA‐3′	95°C‐30 s/60°C‐1 m/72°C‐30 s	40

### 2.2 Culture of Cells

The ARPE‐19 retinal pigment epithelial cell line was cultured in Dulbecco's Modified Eagle's Medium (DMEM) supplemented with 10% fetal bovine serum (FBS), 5% L‐glutamine and 1% penicillin–streptomycin. Culturing conditions involved maintaining the cells at 37°C in a humidified environment with 5% CO_2_ and 100% humidity [19, 20]. The ARPE‐19 cells were used at passages 5–10. Cells were seeded at a density of 1 × 10^4^ cells/well for IC_50_ determination and 5 × 10^4^ cells/well for ELISA and RT‐PCR experiments.

### 2.3 Determination of IC_50_ Doses With MTT Cell Viability Test

Cells were plated in 96‐well plates at 10^4^ cells per well and then treated with OLP, HSP, PIP, PACs and RA at diverse concentrations (1, 10, 25, 50, 100, 250, 500 and 1000 μM) for 48 h including control groups. After the addition of 10 μL of MTT solution (5 mg/mL, SERVA, Heidelberg, Germany) to each well and incubation for 4 h at 37°C with 5% CO_2_, 100 μL of dimethyl sulfoxide (DMSO) was supplemented to each well before measuring their absorbance at 570 nm through an automatic multiplate reader (Epoch, Biotek, USA). IC_50_ values were calculated using GraphPad Prism version 8.0.1 (GraphPad Software Inc., CA, USA). Since BVZ is not toxic to cells, IC_50_ dose calculation was not made and it was administered at a dose of 1 mg/mL based on results reported in literature [21]. Each analysis was performed in triplicate.

### 2.4 Analyses of Total Protein Derived From Cell Lysates

PBS was used to wash the cells twice before harvesting them by lysis buffer including protease inhibitor cocktail (Roche Complete, Indianapolis, USA). Lysates were, then, processed with a centrifuge at 16000 g for 15 min at 4°C. The BCA method (TaKaRa, Shiga, Japan) was used to determine the concentration of protein collected from the supernatant.

### 2.5 ELISA Analyses

Cells underwent a 48‐h treatment with specific agents, followed by incubation. Subsequently, levels of VEGFA and VEGFR1 were quantified in protein extracted from cell lysate using the ELISA technique, adhering to the manufacturer's guidelines. The outcomes were reported as pg/mg protein.

### 2.6 Polymerase Chain Reaction

After completion of the 48‐h incubation, harvesting and washing of cells with cold PBS was accomplished. GeneJET RNA Purification Kit (Thermo Scientific Catalogue No: K0731) was used to isolate total RNA for mRNA expression level and the Epoch Take3 plate system (Agilent, USA) was utilised to analyse the quantity and purity of isolated RNAs. Then, the synthesis of complementary DNA (cDNA) was performed following the instructions of the manufacturer (Biorad Cat No: BR1708891). A template of 1 μg total RNA was selected for the PCR reaction that was executed by reverse transcriptase (RT). After this, 1 μL of cDNA was taken from individual samples and samples were supplemented with SYBR green PCR Master Mix according to the forward and reverse primer protocols. All stages were carried out under cold chain and sterile conditions. Normalisation of expression levels of target genes was made by housekeeping gene GAPDH. Calculation of gene expression values was performed according to the ΔΔCt method following the RQ = 2^−ΔΔ*C*^_t_ equation and using REST2009 program. Table 1 reports the Primer sequences utilised to accomplish the PCR reactions together with the PCR conditions. Each assay was performed in quadruplicate.

### 2.7 Statistical Analysis

GraphPad Prism version 8.0.1 (GraphPad Software Inc., CA, USA) program was used to analyse the data. Normal distribution of data was validated using the Kolmogorov–Smirnov test. Since the data showed normal distribution, the parametric One‐Way ANOVA test was performed for comparison between groups and a significant value of *p* < 0.05 was set as significant.

## 3 Results

### 3.1 IC_50_ Doses of Bioactive Compounds

**FIGURE 2:**
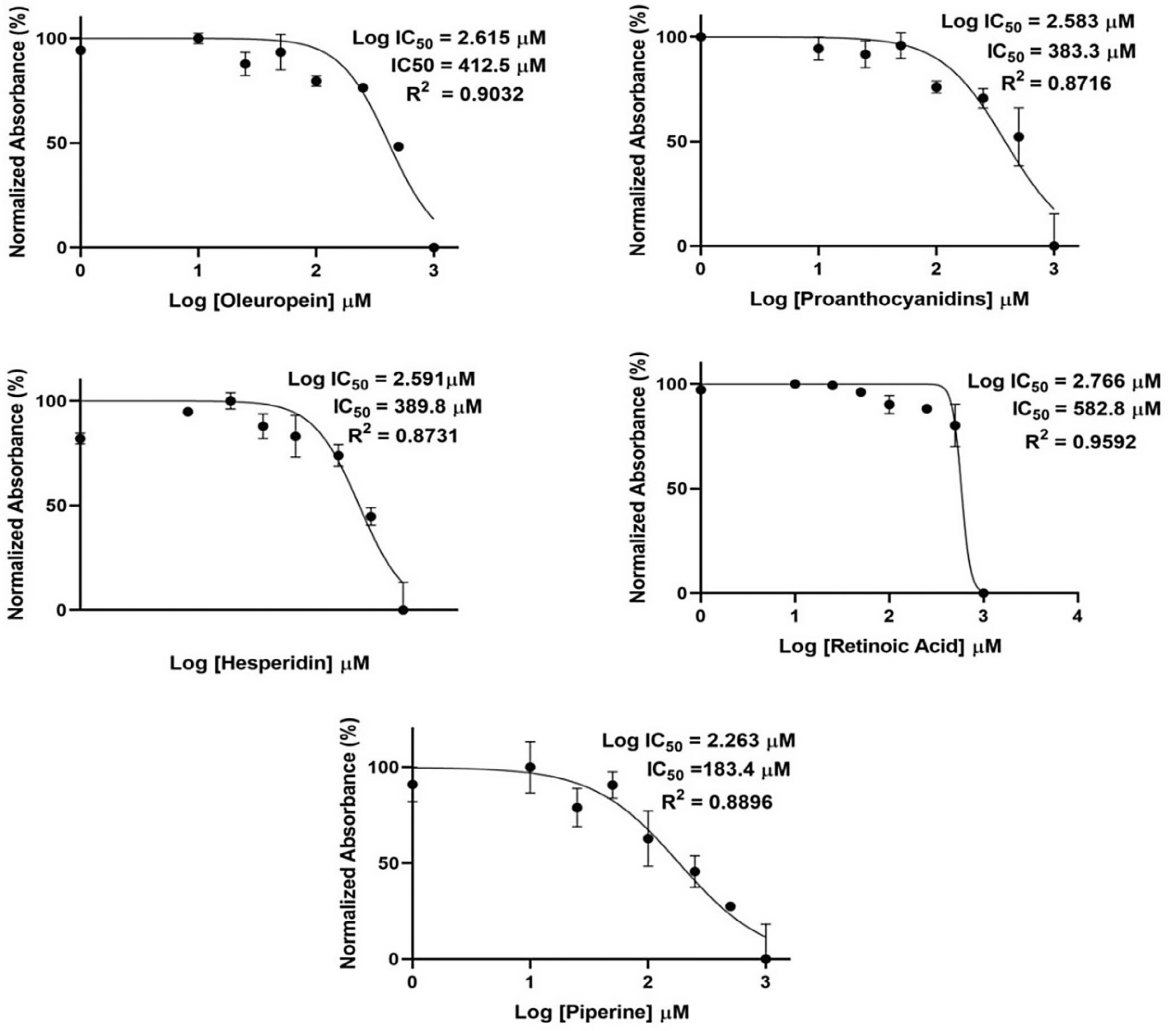
IC_50_ doses of OLP, HSP, PIP, PACs and RA.

As a result, IC_50_ value of OLP was 412.5 μM, HSP was 389.8 μM, PIP was 183.4 μM, PACs was 383.3 μM and RA was 582.8 μM for 48 h treatment (Figures 1 and 2). IC_50_ values were calculated using nonlinear regression analysis with a sigmoidal dose–response curve fitting (GraphPad Prism 8.0.1), based on three independent experiments with triplicate wells for each concentration.

### 3.2 ELISA Findings

VEGFR1 levels were 0.833 ± 0.009 pg/mg protein in the control group, 0.833 ± 0.009 pg/mg in OLP, 0.312 ± 0.001 pg/mg in HSP, 0.352 ± 0,086 pg/mg in PIP, 0.540 ± 0.004 pg/mg in PACs, 0.419 ± 0.105 pg/mg in RA and 0.180 ± 0.002 pg/mg in BVZ (Table 2).

**TABLE 2:** Comparison of VEGFR1 and VEGFA protein levels compared to the control group.

Groups	VEGFR1		VEGFA	
	Levels (pg/mg protein)	*p*	Levels (pg/mg protein)	*p*
Cont	0.363 ± 0.004		0.1003 ± 0.003661	
OLP	0.833 ± 0.009	< 0.0001	0.09768 ± 0.0009	0.6926
HSP	0.312 ± 0.001	0.7259	0.1119 ± 0.001292	0.0006
PIP	0.352 ± 0.086	0.9996	0.08887 ± 0.002542	0.0007
PACs	0.54 ± 0.04	0.0066	0.07695 ± 0.002740	< 0.0001
RA	0.419 ± 0.105	0.6489	0.1305 ± 0.004353	< 0.0001
BVZ	0.18 ± 0.002	0.0048	0.03590 ± 0.001320	< 0.0001

VEGFA levels were 0.1003 ± 0.003661 pg/mg protein in the control group, 0.09768 ± 0.0009 pg/mg in OLP, 0.1119 ± 0.001292 pg/mg in HSP, 0.08887 ± 0.08887 pg/mg in PIP. In addition, 0.002542 pg/mg protein, 0.07695 ± 0.00274 pg/mg in PACs, 0,1305 ± 0.004353 pg/mg in RA, and 0.18 ± 0.002 pg/mg in BVZ (Table 2). VEGFR1 levels were significantly lower in the BVZ group (*p* < 0.01) compared to controls. VEGFA levels in the RA‐treated group were significantly higher than control (*p* < 0.05), while PACs and PIP groups showed significant reductions (*p* < 0.05).

### 3.3 RT‐PCR Findings

In the OLP group, mRNA expression levels of EGF, EGFR, PDGF‐β, PDGFR‐β and HIF1A genes were found to be 0.726, 1.235, 0.329, 1.002 and 0.623, respectively, when compared to the control group. mRNA expression levels of the respective genes in HSP group were determined to be 0.571, 0.931, 0.248, 0.754 and 0.484 compared to the control group. In the PIP group, mRNA expression levels of the respective genes were found to be 0.798, 1.627, 0.491, 0.829 and 0.372 compared to control. mRNA expression levels of the respective genes in the PACs group were determined to be 1.017, 0.746, 0.513, 1.359 and 0.254 compared to the control group. In the BVZ group, mRNA expression levels of the respective genes were found to be 1.057, 1.4, 1.768, 1.125 and 1.359 compared to control (Table 3).

**TABLE 3:** Comparison of mRNA Expression Levels of Genes.

Genes	OLP		HSP		PIP		PACs		RA		BVZ	
	Exp.	*p*	Exp.	*p*	Exp.	*p*	Exp.	*p*	Exp.	*p*	Exp.	*p*
EGF	0.726	0.019	0.571	0.021	0.798	0.015	1017	0.656	1575	< 0.001	1057	0.812
EGFR	1235	0.154	0.931	0.595	1627	0.012	0.746	0.082	0.044	0.019	1400	0.066
PDGF	0.329	0.017	0.248	0.017	0.491	0.009	0.513	0.017	0.236	0.015	1768	0.058
PDGFRß	1002	0.951	0.754	0.036	0.829	0.189	1359	0.080	1729	0.025	1125	0.382
HIF1A	0.623	0.025	0.484	0.016	0.372	0.019	0.254	0.023	0.308	0.016	1359	0.086

**FIGURE 3:**
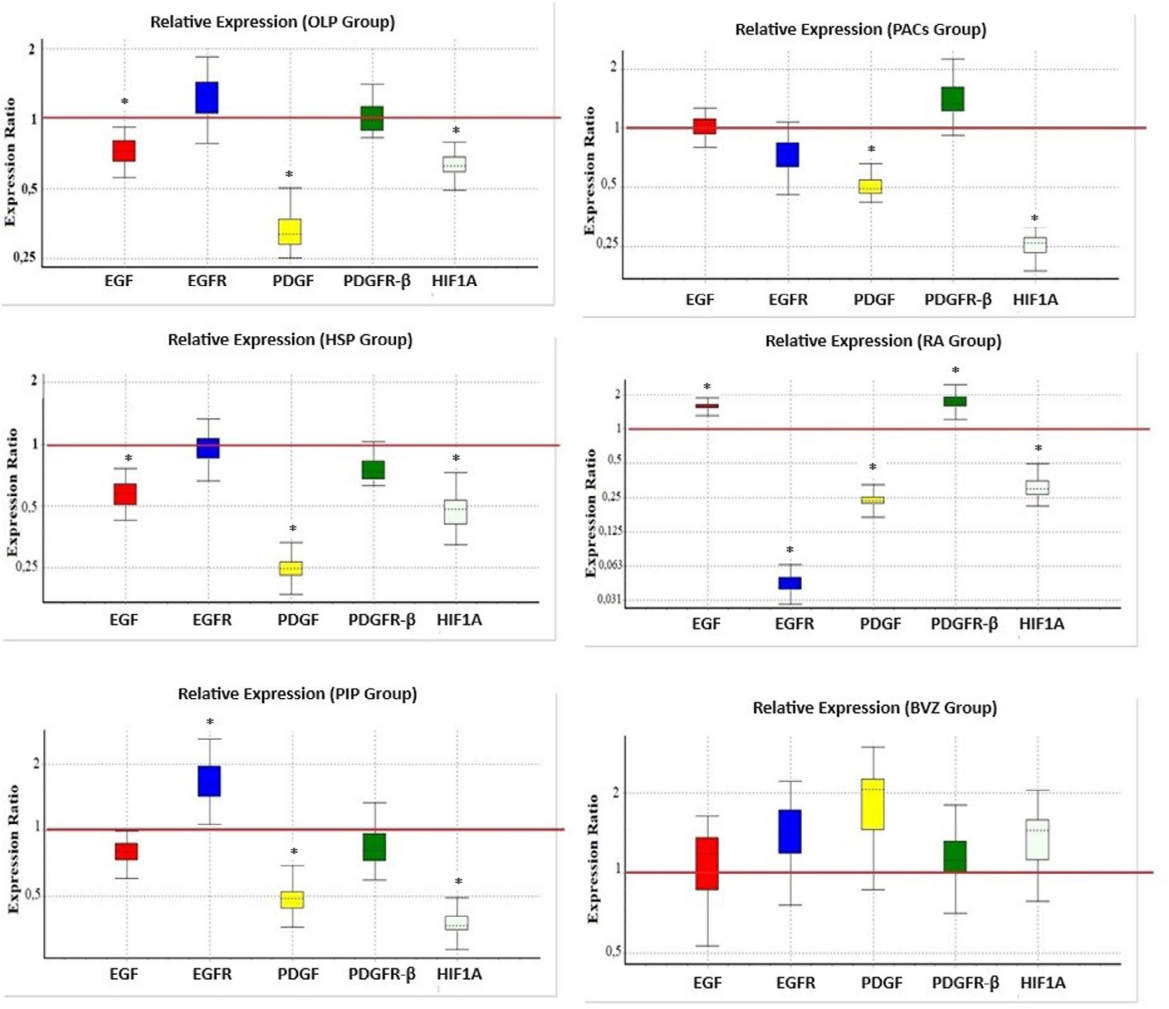
Comparison of relative mRNA expression levels of genes by REST 2009 software (Qiagen). Values are expressed as the mean ± SD. Groups were compared with the control group (red parallel line) and results were given as fold increase/decrease (*p < 0.05).

**FIGURE 4:**
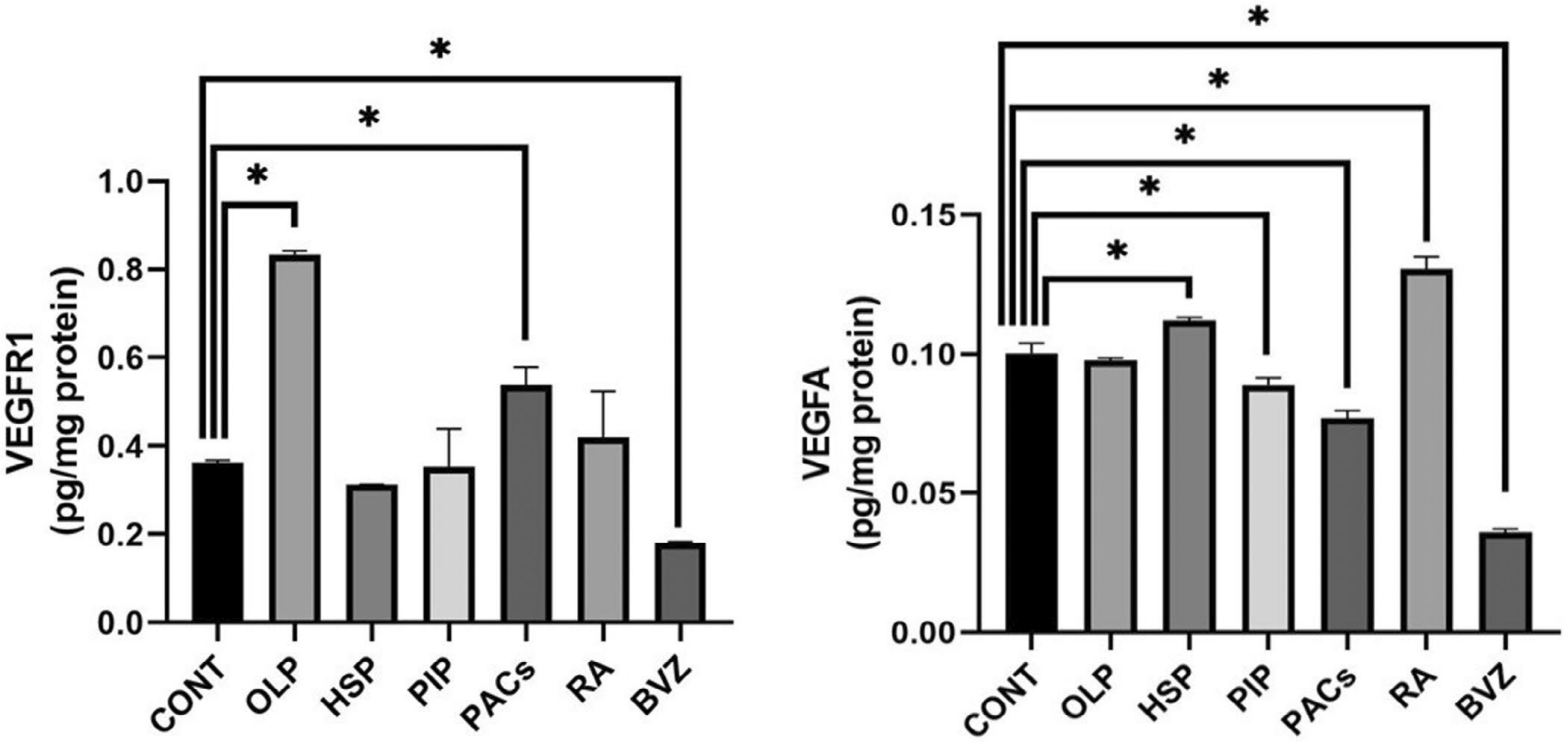
VEGFR1 and VEGFA protein levels comparison between groups (*p < 0.05).

Among all groups, the lowest values in both VEGFA (*p* < 0.05) and VEGFR‐1 (*p* < 0.05) were found in BVZ (Figures 3 and 4). While HSP (*p* > 0.05), PIP (*p* > 0.05) and RA (*p* > 0.05) groups gave results similar to the control group in terms of VEGFR‐1 levels that was highest in the OLP (*p* < 0.05) and PACs (*p* < 0.05) groups compared to the control group.

### 3.4 PACs and PIP Were the Bioactive Compounds That Best Reduced VEGFA Protein Levels After BVZ

PACs significantly decreased VEGFA protein levels compared to the control group (*p* < 0.05) but increased VEFGR‐1 levels (*p* > 0.05). It also suggested an anti‐angiogenic effect through alternative pathways by significantly decreasing the mRNA expression levels of PDGFβ (*p* < 0.05), and HIF1A (*p* < 0.05) and not significantly for PDGFR‐β (*p* > 0.05) and EGFR (*p* > 0.05), compared to the control group. PIP significantly reduced VEGFA protein levels compared to the control group (*p* < 0.05). In addition, PIP showed a significant extra anti‐angiogenic effect from BVZ by reducing the expression levels of EGF (*p* < 0.05), PDGF (*p* < 0.05) and HIF1A (*p* < 0.05).

### 3.5 RA Has an Angiogenic Effect by Increasing VEGFA Protein Levels and Also by Increasing EGF and PDGFR‐β mRNA Expression Levels

**FIGURE 5:**
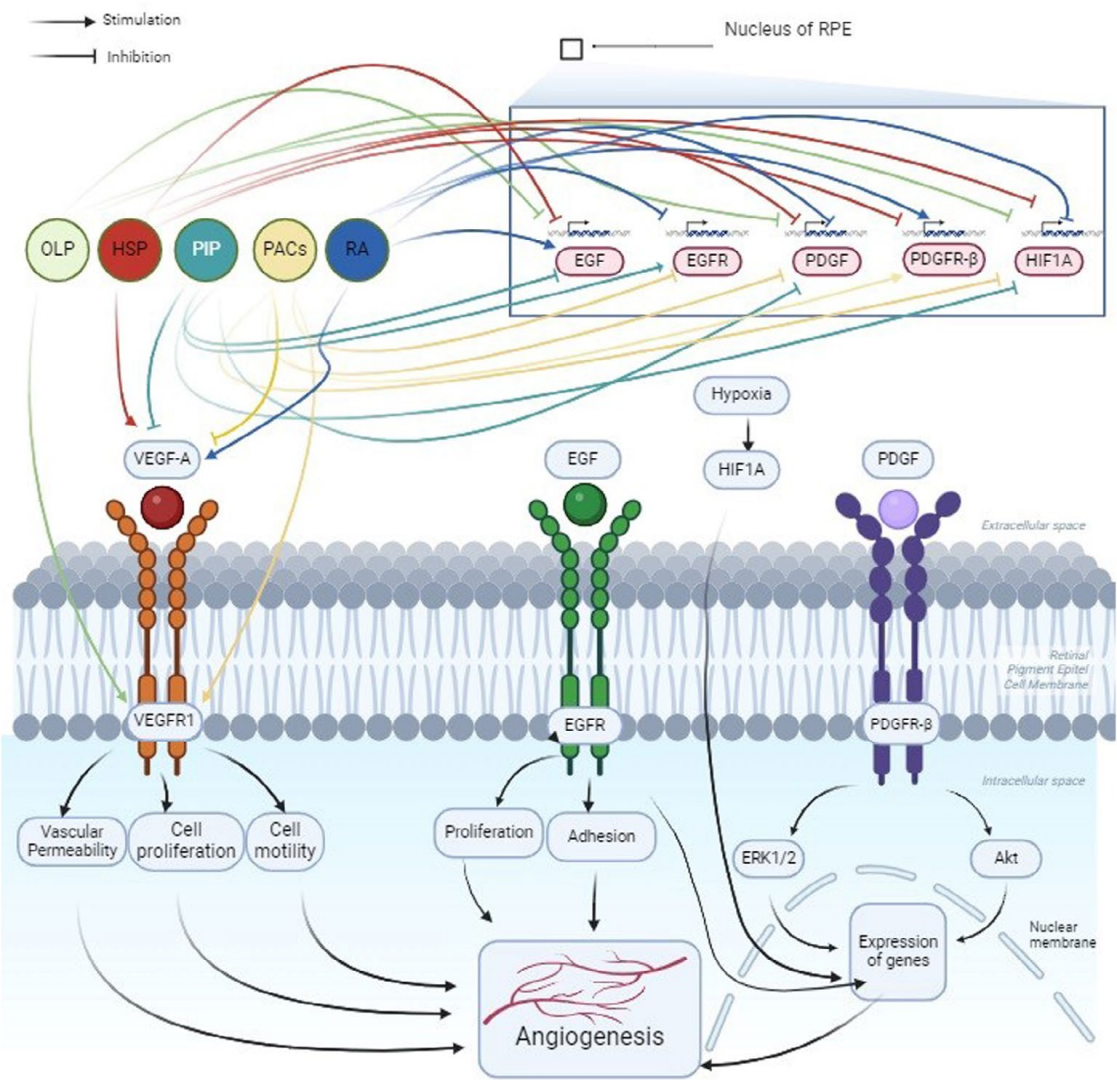
Effects of OLP, HSP, PIP, PACs and RA as bioactive components on angiogenesis in retinal pigment epithelial cells. EGF, Epidermal Growth Factor; EGFR, Epidermal Growth Factor Receptor; HIF1A, Hypoxia‐Inducible Factor‐1α; HSP, Hesperidin; OLP, Oleuropein; PACs, Proantocyanids; PDGF, Platelet Derived Growth Factor; PDGFR, Platelet Derived Growth Factor Receptor Beta; PIP, Piperin; RA, Retinoic Acid; VEGFA, Vascular Endothelial Growth Factor A; VEGFR, Vascular Endothelial Growth Factor Receptor.

HSP (*p* < 0.05) and RA (*p* < 0.05) both significantly increased the VEGFA levels when compared to the control group. Although HSP increases VEGFA levels, it significantly decreases the mRNA expression levels of EGF (*p* < 0.05), PDGFβ (*p* < 0.05) and HIF1A (*p* < 0.05) and not significantly that of PDGFR‐β (*p* > 0.05) compared to the control group. This suggests that it may have a suppressive effect through various pathways on angiogenesis, in retinal pigment epithelial cells. However, in addition to increasing VEGFA levels, RA also demonstrated an angiogenic effect by significantly increasing EGF (*p* < 0.05) and PDGFR‐β (*p* < 0.05) mRNA expression levels. These findings were found to be compatible with results of other studies regarding the presence of RA required for healthy angiogenesis and that angiogenesis is increased in the presence of RA [22, 23] (Figures 3 and 5).

### 3.6 OLP Has an Anti‐Angiogenic Effect on Retinal Pigment Epithelial Cells via an Alternative Pathway

Although OLP significantly increased VEGFR‐1 protein levels (*p* < 0.001), no significant effects were detected on VEGFA protein levels (*p* > 0.05). In addition, OLP showed an anti‐angiogenic property by significantly reducing the mRNA expression levels of EGF (*p* < 0.05), PDGFβ (*p* < 0.05) and HIF1A (*p* < 0.05) (Figure 4).

## 4 Discussion

Angiogenesis is a multifaceted process characterised by numerous stages and various ligand‐receptor interactions, with VEGF playing a pivotal role in regulating almost all these processes. During tumour growth, VEGF is secreted by the tumoural cells in response to diverse stimuli occurring around their microenvironment, including cytokines (such as EGF, FGF, IGF, PDGF), low pH, hypoxia and hypoglycaemia [24, 25]. The secreted VEGF then binds to and activates endothelial cells (EC) in nearby blood vessels. Activated ECs quickly undergo transformations including amplified permeability of macromolecules, NO expression and production of various genes. ECs divide as a reaction to VEGF and other tumour‐derived growth factors. Consequently, new capillaries are shaped and linked to other developed vascular networks creating a capillary network called neovascularisation that is more prone to bleeding [26, 27, 28].

BVZ (Avastin, Genentech) is a humanised anti‐VEGF monoclonal antibody that has been reported to show remarkable effectiveness in suppressing the growth of tumour in different animal models [29] and directly exhibits anti‐vascular effects in colorectal cancers [30]. Importantly, BVZ, when combined with standard chemotherapies, leads to a notable clinical improvement in the survival of colorectal cancer patients [31]. Thus, it has been demonstrated that the anti‐angiogenesis strategy can serve as an effective cancer treatment. Besides the previously demonstrated in vivo efficacy, this study focused on deep investigation to evaluate the anti‐VEGF activities of BVZ by using in vitro models. In all models tested in the study, BVZ showed potent inhibitor activity able to neutralise all VEGF‐mediated activities.

In this study, five different bioactive compounds together with BVZ were analysed in terms of their anti‐angiogenic properties. None of the five different bioactive compounds whose anti‐angiogenic effects were experimentally tested had anti‐VEGFA effects as much as BVZ. However, other bioactive compounds have been shown to have the potential to exert anti‐angiogenic effects through alternative pathways. In line with previous studies, the results of in vitro analyses of this research reported that BVZ is the agent that had the best decrease in both VEGFA and VEGFR‐1 levels among all groups [32, 33].

BVZ is the first molecule that has been approved as an anti‐angiogenic for human cancer and several other VEGF inhibitors are being tested and clinically trailed for this aim. This research proved the strongest inhibition/neutralisation properties of BVZ against VEGF when compared to other VEGF inhibitors and other bioactive molecules studied in various in vitro models.

It should not be ignored that the dramatic reduction of VEGFR‐1 by BVZ, which is emphasised in recent literature as a requested step for normal haematopoiesis [34], may have a negative effect on the haematopoietic compartments. While PACs increase VEGFR‐1 levels, they are the group that better decreases VEGFA levels after BVZ. This means that it may be beneficial to combine PACs with BVZ in order to ensure that normal haematopoiesis continues unaffected and to preserve the anti‐angiogenic effect. Although there is no haematopoietic compartment in the eye, it is important that normal haematopoiesis is not affected by systemic intake of these agents.

Oleuropein (OLP) is known for its anti‐inflammatory, antioxidant, anticancer, cardioprotective, neuroprotective and hepatoprotective effects [10, 35, 36, 37, 38]. In addition, recent trials reported that OLP is able to inhibit proliferation and induce apoptosis in different cancer cell lines [39]. Additionally, researchers on animals have reported that OLP possesses anticancer effects linked to its ability to act as a gene expression and modulator of activity for different signalling proteins involved in proliferation and apoptosis. Although OLP is frequently mentioned as an EGFR‐2 inhibitor in literature [40], in this study, the OLP group showed EGFR mRNA expression levels similar to the control group. Additionally, a reduction in the mRNA expression levels of PDFGB [41, 42], an angiogenic molecule, was reported in the OLP group. This is the first study reporting that OLP causes PDGFB reduction in retinal pigment epithelial cells (Figure 5).

Kunimi et al. [43] reported that silenced HIF1A gene in mice showed less inner retinal degeneration than in control group. Additionally, it has been previously reported that downregulation of HIF1A has a very important role to prevent neovascularisation in the retina [44]. Another important finding in this study is that all experimentally tested bioactive compounds reduced HIF1A mRNA expression levels, except for the BVZ group.

HSP belongs to the flavanone class of flavonoids and possesses wide applicability in prevention of cardiovascular, neurodegeneration and cancer diseases. HSP anticancer effects are related to its anti‐inflammatory and anti‐oxidant activities [45, 46, 47, 48, 49, 50, 51, 52, 53]. HSP interacts with multiple well‐known cellular targets and it is able to inhibit the proliferation of cancer cells due to induction of apoptosis and stop cell cycle. Additionally, it is suggested that it plays an encouraging part in inhibition of tumour cell metastasis, angiogenesis and chemoresistance.

In this study, although HSP increased VEGFA levels, it suppressed angiogenesis in retinal pigment epithelial cells through alternative pathways by decreasing EGF, PDGFβ, PDGFR‐β and HIF1A mRNA expression levels. This finding is a valuable result proving that angiogenesis is not suppressed only through a single pathway but can also be suppressed through alternative pathways.

Piperine (PIP) has various physiological effects, including that of being able to kill cancer cells, and well‐documented angiogenesis effects [7]. In one study, PIP showed an antiangiogenic effect by inhibiting the phosphorylation of protein kinase B residues, which are the main regulators of endothelial cell function and angiogenesis [7]. In the same study, PIP showed its antiangiogenic effect being able to inhibit the proliferation of human umbilical vein endothelial cells (HUVEC) and collagen‐induced angiogenesis through protein kinase B inhibition. Considering these results, they support additional investigation of PIP as an angiogenesis inhibitor to be applied in cancer treatment. In this study, PIP reduced VEGFA protein levels and also showed a greater anti‐angiogenic effect than BVZ by reducing EGF, PDGF and HIF1A expression levels.

The antiproliferative effect of retinoic acid (RA) was reported for a variety of tumours and the results suggested a connection of these effects from redifferentiation. Currently, only scarce information is available little about the RA effects on the angiogenesis of tumour, that is the precondition leading to its growth and metastatic spread. A previous study indicated that the growth of tumour cells of thyroid was inhibited by RA [8]. They found a decrease in in vitro VEGF growth in thyroid carcinoma and thought that decreased angiogenesis provided therapeutic effect of RA in thyroid cancer [8]. Additionally, they stated that RA has a direct antiproliferative effect on human endothelial cells [8]. However, in the present study, unlike the previous cited research [8], RA showed an angiogenic effect by increasing VEGFA protein levels and also by increasing EGF and PDGFR‐β mRNA expression levels. These results were consistent with those of earlier studies reporting that the presence of RA is required for healthy angiogenesis and that angiogenesis is increased in the presence of RA [19, 20] (Figures 3 and 5).

Proanthocyanidins are tannins possessing several pharmacological properties. PACs were reported to provide significant health benefits, as a result of several researchers involving both human and animal models [11]. The compounds have proven beneficial properties, such as anticancer, antioxidant, antidiabetic, antimicrobial and neuroprotective. In addition, they showed to have positive effects to protect from cardiovascular disorders, metabolic disorders and oncogenic events. Therefore, PACs have been suggested as a potential pharmaceutical agent for the treatment of such diseases [54].

In this study, PACs, along with PIP, were the bioactive compounds that best reduced VEGFA protein levels after BVZ. These results underline the anti‐angiogenic effect of PACs in line with the reports in literature.

Although this study is the first to experimentally test more than one pathway and more than one bioactive compound on angiogenesis in retinal pigment epithelial cells, its biggest limitation was the inability to analyse the protein levels of genes whose mRNA expression levels were measured due to financial constraints.

In this study, only PACs and PIP, among the bioactive compounds whose anti‐angiogenic properties have been previously investigated, were found to have anti‐VEGF and anti‐angiogenic properties. As expected, the anti‐angiogenic properties of BVZ were superior to the other five bioactive compounds. While several previous studies focusing on the effects of these bioactive components on tumorigenesis have been carried out, studies on anti‐VEGF and anti‐angiogenesis are still very limited. This is another factor that makes this study valuable given that here the focus was on the effects of these mentioned bioactive components, particularly on VEGF, which is implicated in the aetiology of age‐related macular degeneration (AMD).

Considering that angiogenesis does not proceed only through VEGF, this study is innovative by investigating and reporting for the first time the effects of relevant bioactive components on angiogenesis through different mechanisms in retinal pigment epithelial cells.

## Author Contributions

**Serkan Sen:** conceptualization (lead), data curation (lead), formal analysis (equal), methodology (equal), resources (equal), validation (equal), visualization (equal). **Murat Kasikci:** conceptualization (equal), data curation (equal), formal analysis (lead), investigation (equal), software (lead), supervision (lead), writing – original draft (lead), writing – review and editing (lead).

## Conflicts of Interest

The authors declare no conflicts of interest.

## Data Availability

All data generated or analysed during this study are included in this published article. The datasets generated during and/or analyzed during the current study are available in the article repository, accessible via https://doi.org/10.1111/jcmm.70327.
